# Smoking and smoking cessation among smokers in Saudi Arabia: A cross-sectional study

**DOI:** 10.18332/tid/208066

**Published:** 2025-09-12

**Authors:** Hassan Arida, Abdulaziz A. Alhothali, Thamer H. AlOtaibi, Osama N. Almalki, Anas H. Alosaimi, Abdullah T. Alenazi, Abdulrahman A. Al Boqami, Ahmed S. Eldalo, Majed A. Algarni, Musaad Althobaiti, Sayed F. Abdelwahab

**Affiliations:** 1 Department of Pharmaceutical Chemistry, College of Pharmacy, Taif University, Taif, Saudi Arabia; 2 College of Pharmacy, Taif University, Taif, Saudi Arabia; 3 Department of Clinical Pharmacology, Clinical Pharmacy University of Palestine, Gaza, Palestine; 4 Department of Clinical Pharmacy, College of Pharmacy, Taif University, Taif, Saudi Arabia; 5 Department of Pharmacology and Toxicology, College of Pharmacy, Taif University, Taif, Saudi Arabia; 6 Department of Pharmaceutics and Industrial Pharmacy, College of Pharmacy, Taif University, Taif, Saudi Arabia

**Keywords:** smoking, cross-sectional, smoking control, Saudi Arabia

## Abstract

**INTRODUCTION:**

This study assessed the general perceptions of smokers in Saudi Arabia about the harms of smoking and identified the different methods used by them to quit smoking.

**METHODS:**

A descriptive cross-sectional study was designed using an online self-administered questionnaire to assess the smoker’s perceptions on smoking and ways to quit smoking. A convenience sample of 1358 participants aged ≥18 years were enrolled from December 2022 to February 2023. Data analysis was carried out using the Statistical Package for Social Sciences (SPSS) software with p<0.05 indicating statistical significance.

**RESULTS:**

A total of 1358 participants completed the online survey. Most of the respondents were smokers (63.3%; n=860). Forty-six percent of the participants (n=396) started smoking when they were aged <18 years. Around 71% of the participants did not like the presence of any family member who smokes. More than half (52%) of the participants admitted that friends are the main reason for beginning smoking. Around 40% (n=229) of the 860 participants were enrolled in a smoking cessation program at some point.

**CONCLUSIONS:**

Around 46% of the study participants started smoking when they were aged <18 years. About two-thirds of them tried to quit smoking. Although many centers around the country are dedicated to helping smokers quit smoking, many smokers noted that their outreach should be further enhanced.

## INTRODUCTION

Cigarettes are the most popular method for consuming tobacco, but there are also vaping devices, cigars or pipes, and storing or chewing tobacco in the mouth^1-3^. While the damages are due to burning tobacco, two forms of smoke come from this burning. The first form is mainstream smoke, the smoke that the person exhales, and the second form is the side stream smoke, which is the smoke from the burned end of a cigarette, pipe, or cigar. This type of smoke has the highest concentration of nicotine and carcinogens^4^.

The most reported risk factors included the social influence of friends, teachers, or parents, low academic performance, having free time, living away from families, and the desire to relieve stress and anxiety from smoking^5^. Interestingly, smokers may argue that the ongoing stress they experience in their daily lives is one of the reasons they are unable to stop smoking. However, evidence of a definite relationship between stress and the urge to smoke is limited^6^. Another factor that affects the decision to smoke is the smoking status of family and close friends and their influence on the smoker, especially adolescents^7^. Moreover, smoking is one of the main causes of different diseases, e.g. cancer^8^. In this regard, smoking is associated with inhibiting the natural immune system that contributes to the development of cancer cell progression^9,10^.

In the world, more than 80% of smokers live in low- and middle-income countries. According to the 2020 statistics, 22.3% of the world’s population uses tobacco, including 36.7% of men and 7.8% of women worldwide^11^. In Saudi Arabia, the percentage of smokers among men reached 30% while it was only 4% among women, with a smoking rate of 14.3% in those aged ≥15 years, excluding smokeless tobacco^11^.

This study aimed to assess the general perceptions of smokers in Saudi Arabia, their readiness to quit, and the available methods to quit smoking.

## METHODS

### Study settings and participants

A descriptive cross-sectional study among Saudi residents was conducted using convenience sampling. The study was conducted from December 2022 to February 2023. Volunteers aged ≥18 years who live in Saudi Arabia participated in the study, while children and those residing in other countries were excluded. Taif University Ethical Committee approved the study protocol (Approval # 44-126). The survey link was distributed on several social media platforms, such as Telegram, Twitter, and WhatsApp groups, with a brief description of the study’s aim, and the participation was voluntary. Consent to participate in the study and that the participant is aged ≥18 years was obtained online at the beginning of the survey. Responses were limited to one by a single individual in the Google form settings. The data obtained were collected from foreigners and citizens, including men and women. A total of 1358 participants successfully submitted the online survey, and the responses were kept on a password-protected Google Drive.

### Study tool and measures

The study tool was a self-administered online questionnaire designed after consulting previously published literature. The questionnaire contained demographic data in addition to 28 questions that assessed the knowledge, attitudes, and general perceptions of smokers in Saudi Arabia about the harms of smoking and different ways to quit it. The questionnaire was prepared in English and translated into Arabic for upload on a Google form for implementation. The questionnaire consisted of the following four sections:

*Section 1*: Participants were asked to indicate their demographics, such as gender, age, residency city/area, education level, marital status, employment, presence of chronic diseases, monthly salary, and smoking status.

*Section 2*: This section contained 13 questions determining the participants’ perception of smoking.

*Section 3*: This section consisted of 10 questions to assess the participants’ perception of quitting smoking.

*Section 4*: This section contained five questions to identify methods used to quit smoking.

### Statistical analysis

Descriptive statistics were generated for the responses and correlation coefficients to describe relationships between continuous variables. For independent variables, the chi-squared test was applied to compare categorical variables, and a p<0.05 was considered significant. Statistical analyses for the gathered data were performed using the Statistical Package for Social Sciences software, version 25 (SPSS, IBM Corporation, NY, USA).

## RESULTS

### Demographic characteristics of the study participants

The 1358 respondents’ demographic characteristics are summarized in Table 1. Most participants were males (n=984; 72.5%). The data show that those who live in Taif were 595 (43.9%), Makkah 155 (11.4%), and Jeddah 321 (23.7%). Regarding education, the percentage of participants whose education level was university student or degree was 878 (74.6%), while that of illiteracy was 16 (1.2%). In general, the economic level of the respondents is relatively high, as it was found that 57.5% had an income of more than 3000 Saudi Riyals. The data showed that 941 (69.3%) participants do not suffer from any chronic disease. Regarding smoking status, 860 (63.3%) of the sample were smokers, of whom 86.7% were males.

**Table 1:** Demographic characteristics of the study participants (N=1358)

*Characteristics*	*Category*	*n (%)*
**Gender**	Male	984 (72.5)
	Female	374 (27.5)
**Age** (years)	<25	560 (41.2)
	26–40	550 (40.5)
	>40	248 (18.3)
**City of residence**	Taif	595 (43.9)
	Makkah	155 (11.4)
	Jeddah	321 (23.7)
	Yanbu	71 (5.2)
	Madinah	89 (6.6)
	Other	127 (9.1)
**Education level**	Uneducated	16 (1.2)
	Primary or intermediate	63 (4.6)
	High school	294 (21.6)
	University student/Bachelor’s degree	878 (74.6)
	Higher education	107 (7.9)
**Marital status**	Single	743 (54.7)
	Married	514 (37.8)
	Divorced	80 (5.9)
	Widow	21 (1.6)
**Employment status**	Not working now	223 (16.4)
	Student	477 (35.1)
	Government employee	419 (30.9)
	Employee in the private sector	239 (17.6)
**Diseases**	None	941 (69.3)
	Obesity	88 (6.5)
	Diabetes	75 (5.5)
	Hypertension	52 (3.8)
	Respiratory diseases (such as asthma)	86 (6.3)
	Immune disorders	11 (0.8)
	Other	105 (9.9)
**Monthly income** (SAR)	<3000	577 (42.5)
	3000–10000	456 (33.6)
	>10000	325 (23.9)
**Smoking**	Yes	860 (63.3)
	No	498 (36.7)

SAR: 1000 Saudi Riyals about US$270.

### Assessment of the smokers’ perception of smoking

The attitude of the smokers who participated in the study is shown in Table 2. Nearly half (52.6%; n=452) of the 860 smokers started smoking when they were aged between 18 and 30 years, and this was significantly associated with male gender (p=0.028). About 38.1% (n=328) of smokers consumed between 11 to 20 cigarettes per day. As shown in Table 2, approximately 61% (n=524) of the 860 smokers reported that one of their family members smoked, while 71.2% (n=612) of smokers did not like that someone in their family smokes. Moreover, 66.3% (n=570) of the smokers strongly agreed/agreed that having friends who smoke is the primary cause of smoking. Furthermore, 57.7% (n=496) of smokers strongly disagreed/disagreed that family is the primary cause of smoking. About 77% of the smokers did not take any chronic disease medications.

**Table 2:** Saudi smokers’ characteristics and perceptions of smoking, by gender (N=860)

*Items*	*All* *n (%)*	*Males* *n (%)*	*Females* *n (%)*	*p*
**How long have you been smoking?** (years)				
<5	297 (34.5)	220 (25.6)	77 (9)	<0.001*
5–15	398 (46.3)	369 (42.9)	29 (3.4)	
16–30	125 (14.5)	119 (13.8)	6 (0.7)	
>30	40 (4.7)	38 (4.4)	2 (0.2)	
**How old were you when you first started smoking?** (years)				
<18	396 (46.0)	353 (41.0)	43 (5)	0.028*
18–30	452 (52.6)	385 (44.8)	67 (7.8)	
>30	12 (1.4)	8 (0.9)	4 (0.5)	
**How many cigarettes do you smoke per day?**				
≤10	232 (27.0)	173 (20.1)	59 (6.9)	<0.001*
11–20	328 (38.1)	290 (33.7)	38 (4.4)	
21–30	237 (27.6)	223 (25.9)	14 (1.6)	
>30	63 (7.3)	60 (7)	3 (0.3)	
**Do you smoke in front of your family?**				
Yes	302 (35.1)	280 (32.6)	22 (2.6)	<0.001*
No	558 (64.9)	466 (54.2)	92 (10.7)	
**Is there any member of your family who smokes?**				
Yes	524 (60.9)	463 (53.8)	61 (7.1)	0.081
No	336 (39.1)	283 (32.9)	53 (6.2)	
**I accept that someone in my family smokes**				
Strongly agree	16 (1.9)	12 (1.4)	4 (0.5)	0.340
Agree	60 (7.0)	49 (5.7)	11 (1.3)	
Neutral	172 (20.0)	147 (17.1)	25 (2.9)	
Disagree	275 (32.0)	244 (28.4)	31 (3.6)	
Strongly disagree	337 (39.2)	294 (34.2)	43 (5)	
**Friends are the main reason for smoking**				
Strongly agree	298 (34.7)	253 (29.4)	45 (5.2)	0.251
Agree	272 (31.6)	88 (10.2)	27 (9.9)	
Neutral	141 (16.4)	118 (13.7)	23 (2.7)	
Disagree	99 (11.5)	245 (28.5)	11 (1.3)	
Strongly disagree	50 (5.8)	42 (4.9)	50 (5.8)	
**Family is the main reason for smoking**				
Strongly agree	26 (3.0)	22 (2.6)	114 (13.3)	0.011*
Agree	138 (16.0)	109 (12.7)	29 (3.4)	
Neutral	200 (23.3)	169 (19.7)	31 (3.6)	
Disagree	246 (28.6)	224 (26)	22 (2.6)	
Strongly disagree	250 (29.1)	222 (25.8)	28 (3.3)	
**Imitation of likable characters is the main reason for smoking**				
Strongly agree	100 (11.6)	75 (8.7)	25 (2.9)	0.004*
Agree	247 (28.7)	215 (25)	32 (3.7)	
Neutral	188 (21.9)	167 (19.4)	21 (2.4)	
Disagree	186 (21.6)	162 (18.8)	24 (2.8)	
Strongly disagree	139 (16.2)	127 (14.8)	12 (1.4)	
**Were any of your friends or family members influenced by you and started smoking?**				
Yes	129 (15.0)	111 (2.9)	18 (2.1)	0.186
No	368 (42.8)	328 (38.1)	40 (4.7)	
I don’t know	363 (42.2)	307 (35.7)	56 (6.5)	

* Significant p<0.05.

### Smokers’ perception of quitting smoking

Table 3 presents the smokers’ perceptions of quitting smoking, where 74% (n=636) of the 860 smokers reported that a smoking cessation service was available in their location. Also, 74.3% (n=639) of the smokers admitted that schools, universities, or other governmental entities prohibit smoking on their campus. In addition, around 88% (n=756) of the smokers supported obligating strict anti-smoking regulations.

**Table 3:** Saudi smokers’ perceptions of quitting smoking (N=860)

*Items*	*All* *n (%)*	*Males* *n (%)*	*Females* *n (%)*	*p*
**Is there a smoking cessation service in your location?**				
Yes	636 (74.0)	560 (65.1)	76 (8.8)	0.114
No	52 (6.0)	45 (5.2)	7 (0.8)	
NA	172 (20.0)	141 (16.4)	31 (3.6)	
**Does school, university or other governmental entity prohibit smoking in its campus?**				
Yes	639 (74.3)	559 (65)	80 (9.3)	0.293
No	103 (12.0)	90 (10.5)	13 (1.5)	
NA	118 (13.7)	97 (11.3)	21 (2.4)	
**Do you support obligating harsh anti-smoking regulations?**				
Yes	756 (87.9)	660 (76.7)	96 (11.2)	0.194
No	104 (12.1)	86 (10)	18 (2.1)	
**Do you support imposing strict rules to fight smoking?**				
Yes	513 (59.7)	444 (51.6)	69 (8)	0.838
No	347 (40.3)	302 (35.1)	45 (5.2)	
**Family is the most influential on me to be able to convince me to quit smoking**				
Strongly agree	252 (29.3)	209 (24.3)	43 (5.0)	0.008*
Agree	292 (34.0)	270 (31.4)	22 (2.6)	
Neutral	177 (20.6)	150 (17.4)	27 (3.1)	
Disagree	99 (11.5)	85 (9.9)	14 (1.6)	
Strongly disagree	40 (4.7)	32 (3.7)	8 (0.9)	
**The teacher is the most influential person on me to be able to convince me to quit smoking**				
Strongly agree	104 (12.1)	90 (10.5)	14 (1.6)	0.013*
Agree	121 (14.1)	109 (12.7)	12 (1.4)	
Neutral	286 (33.3)	253 (29.4)	33 (3.8)	
Disagree	205 (23.8)	182 (21.2)	23 (2.7)	
Strongly disagree	144 (16.7)	112 (13)	32 (3.7)	
**A smoker is an outcast in society**				
Strongly agree	51 (5.9)	39 (4.5)	12 (1.4)	0.092
Agree	135 (15.7)	112 (13)	23 (2.7)	
Neutral	248 (28.8)	217 (25.2)	31 (3.6)	
Disagree	298 (34.7)	265 (30.8)	31 (3.6)	
Strongly disagree	128 (14.9)	113 (13.1)	15 (1.7)	
**I find it difficult to stop smoking in public places where it is forbidden, such as in malls**				
Strongly agree	134 (15.6)	117 (13.6)	17 (2.0)	0.001*
Agree	255 (29.7)	237 (27.6)	18 (2.1)	
Neutral	212 (24.7)	169 (19.7)	43 (5.0)	
Disagree	165 (19.2)	140 (16.3)	25 (2.9)	
Strongly disagree	94 (10.9)	83 (9.7)	11 (1.3)	
**Do you want to quit smoking?**				
Yes	697 (81.0)	617 (71.7)	80 (9.3)	0.001*
No	163 (19.0)	129 (15.0)	34 (4.0)	
**Have you tried to quit before?**				
Yes	573 (66.6)	514 (59.8)	59 (6.9)	<0.001
No	287 (33.4)	232 (27)	55 (6.4)	

* Significant p<0.05.

As shown in Table 3, approximately 45% (n=389) of the smokers strongly agreed/agreed that it is difficult to stop smoking in public places where it is forbidden, such as in malls, while only 30.1% (n=259) of the participants strongly disagreed/disagreed with this statement. On the other hand, about 81% (n=697) of the smokers wanted to quit smoking. Also, 66.6% (n=573) of the smokers have tried to quit smoking before.

### Assessment of methods used to quit smoking

The responses of the smoking participants toward the methods used to quit smoking are shown in Table 4. As shown, 40% (n=229) of the 860 participants were enrolled in a smoking cessation program at some point. In addition, 15.7% (n=90) of the participants used nicotine patches that helped them quit smoking, while 7.2% (n=41) of the participants had ever used nicotine lozenges. Overall, 35% (n=200) and 26.7% (n=152) of the participants used one and two to three methods to quit smoking, respectively. As shown in Table 4, about two-thirds of the smokers in the study (62%) tried at least one method to quit smoking.

**Table 4:** Methods used to quit smoking among smokers in Saudi Arabia (N=573)

*Questions*	*Response* *n (%)*		
	*Yes*		*No*
**Did you join any smoking cessation program?**			
All	229 (40.0)		344 (60.0)
Males	213 (37.2)		301 (52.5)
Females	16 (2.8)		43 (7.5)
	Yes, and it helped me to quit	Yes, but it didn’t help me to quit	No
**Have you used CHAMPIX before?**	109 (19)	109 (19)	355 (62)
**Have you used nicotine patches to help quit smoking?**	90 (15.7)	131 (22.9)	352 (61.4)
**Have you used nicotine lozenges to help quit smoking?**	41 (7.2)	64 (11.2)	468 (81.7)
**How many of the above methods have you used in your attempt to quit smoking?**			
1	200 (34.9)		
2	106 (18.5)		
3	46 (8.2)		
I haven’t used any	220 (38.4)		

## DISCUSSION

This study provides an overview of the characteristics of smokers in Saudi Arabia and their experiences in smoking cessation. Of the 1358 respondents, 63.3% were smokers in our sample, the majority of which were male (86.7%), while female smokers represented only a small percentage 13.3%.

Among the participants, 46.3% of the smokers said they had been smoking for five to fifteen years. In this regard, the study performed in Al-Ahsa, Saudi Arabia, found that 44.6% of the smokers had been smoking for six to fifteen years^12^, which is similar to our findings.

Nearly half of the smokers in our study started smoking when they were aged between 18 and 30 years, while the rest of the smoking participants started smoking even before they reached the age of 18 years. Another study conducted in the Jazan region in Saudi Arabia^13^ found that around 66% started smoking before the age of 18 years.

Regarding the number of cigarettes per day, our data revealed that 38.1% of smokers smoke between 11 and 20 cigarettes per day, which is similar to another study conducted in Saudi Arabia^14^, where they found that 34% of smokers smoke between 11 and 20 cigarettes. Also, about 71.2% of our smokers did not accept that someone in their family smokes. This attitude is relatively higher than those obtained in Palestine (50.4%)^15^. Nevertheless, about 66.3% of our smokers strongly agreed/agreed that having bad friends is the primary cause of smoking. Moreover, about 19% of smokers in this study strongly agreed/agreed that family is the primary cause of smoking. This finding is not far away from the finding of another study conducted in Saudi Arabia^16^, where 12.3% of the participants admitted that smokers within the family were their primary motivation for smoking. However, about 42.2% of smokers in our study did not know whether any of their friends or family members were influenced by them and started smoking.

Regarding smoking cessation services, about 74% of smokers in our study said that such services are encountered in their location. On the other hand, about 74.3% of the smokers in our study said that schools, universities, or other governmental entities prohibit smoking in their buildings. This finding agrees with the finding of a systematic review of smokers’ attitudes toward smoking cessation conducted in Saudi Arabia^17^, where the authors concluded that even though governmental buildings, university campuses, hospitals, and other public facilities prohibit smoking, secondhand smoking with family and friends remains problematic.

Regarding the desire of the smoker to quit smoking, the responses of the smokers in our study (81%) are relatively higher than those (70.2%) reported in another study conducted in Saudi Arabia^15^. Nevertheless, about 63.3% of our smokers reported that the family is the most influential in convincing them to quit smoking.

### Strengths and limitations

One of the study’s strengths is its relatively large sample size, another is that it covered the entire country and did not focus on a specific region. One of the limitations is the cross-sectional design which does not allow for causal inferences, while the most important limitation is that the convenience sampling method via social media was non-probability sampling that could lead to selection bias, and thus the results of this study cannot be generalized to the entire population of Saudi smokers. Additionally, as no further statistical analyses were performed on the data, we cannot rule out other factors that may have influenced the perceptions of smokers.

## CONCLUSIONS

Around 46% of the study participants started smoking when they were younger than 18 years. About two-thirds of them tried to quit smoking. Although many centers around the country are dedicated to helping smokers quit smoking, many smokers noted that their outreach should be further enhanced. Despite the inherent limitations of the study, these data could be useful as background information when planning awareness and educational campaigns in Saudi Arabia.

## Data Availability

The data supporting this research are available from the authors on reasonable request.

## References

[1] Miglio F, Naroo S, Zeri F, Tavazzi S, Ponzini E. The effect of active smoking, passive smoking, and e-cigarettes on the tear film: an updated comprehensive review. Exp Eye Res. 2021;210:108691. doi:10.1016/j.exer.2021.108691 34224681

[2] Raupach T, Radon K, Nowak D, Andreas S. Passivrauchen: gesundheitliche folgen, effekte einer expositionskarenz und präventionsaspekte. Pneumologie. 2008;62(1):44-48. doi:10.1055/s-2007-980154 18041691

[3] Centers for Disease Control and Prevention. Consumption of cigarettes and combustible tobacco - United States, 2000-2011. MMWR Morb Mortal Wkly Rep. 2012;61(30):565-569. 22854624

[4] Arastoo S, Haptonstall KP, Choroomi Y, et al. Acute and chronic sympathomimetic effects of e-cigarette and tobacco cigarette smoking: role of nicotine and non-nicotine constituents. Am J Physiol Heart Circ Physiol. 2020;319(2):H262-H270. doi:10.1152/ajpheart.00192.2020 32559135 PMC7473924

[5] Urrutia-Pereira M, Oliano VJ, Aranda CS, Mallol J, Solé D. Prevalence and factors associated with smoking among adolescents. J Pediatr. 2017;93(3):230-237. doi:10.1016/j.jped.2016.07.003 27886805

[6] Schultz ME, Fronk GE, Jaume N, Magruder KP, Curtin JJ. Stressor-elicited smoking and craving during a smoking cessation attempt. J Psychopathol Clin Sci. 2022;131(1):73-85. doi:10.1037/abn0000702 34881919 PMC8979188

[7] Pollard MS, Tucker JS, Green HD, Kennedy D, Go MH. Friendship networks and trajectories of adolescent tobacco use. Addict Behav. 2010;35(7):678-685. doi:10.1016/j.addbeh.2010.02.013 20332061

[8] West R. Tobacco smoking: health impact, prevalence, correlates and interventions. Psychol Health. 2017;32(8):1018-1036. doi:10.1080/08870446.2017.1325890 28553727 PMC5490618

[9] Kondo T, Nakano Y, Adachi S, Murohara T. Effects of tobacco smoking on cardiovascular disease. Circ J. 2019;83(10):1980-1985. doi:10.1253/circj.CJ-19-0323 31462607

[10] Rezk-Hanna M, Benowitz NL. Cardiovascular Effects of hookah smoking: potential implications for cardiovascular risk. Nicotine Tob Res. 2019;21(9):1151-1161. doi:10.1093/ntr/nty065 29660041

[11] Johnston PI, Wright SW, Orr M, et al. Worldwide relative smoking prevalence among people living with and without HIV. AIDS. 2021;35(6):957-970. doi:10.1097/QAD.0000000000002815 33470609

[12] Al Rajeh AM, Mahmud I, Al Imam MH, et al. E-Cigarette use among male smokers in Al-Ahsa, Kingdom of Saudi Arabia: a cross-sectional study. Int J Environ Res Public Health. 2022;20(1):143. doi:10.3390/ijerph20010143 36612462 PMC9819296

[13] Abdelwahab SI, El-Setohy M, Alsharqi A, Elsanosy R, Mohammed UY. Patterns of use, cessation behavior and socio-demographic factors associated with smoking in Saudi Arabia: a cross- sectional multi-step study. Asian Pac J Cancer Prev. 2016;17(2):655-660. doi:10.7314/apjcp.2016.17.2.655 26925659

[14] Al-Zalabani AH, Abdallah AR, Alqabshawi RI. Intention to quit smoking among intermediate and secondary school students in Saudi Arabia. Asian Pac J Cancer Prev. 2015;16(15):6741-6747. doi:10.7314/apjcp.2015.16.15.6741 26434904

[15] Eldalo AS. Prevalence and perception of smoking habits among the Palestinian population in the Gaza Strip. J Multidiscip Healthc. 2016;9:297-301. doi:10.2147/JMDH.S107346 27486330 PMC4956068

[16] Baig M, Bakarman MA, Gazzaz ZJ, et al. Reasons and motivations for cigarette smoking and barriers against quitting among a sample of young people in Jeddah, Saudi Arabia. Asian Pac J Cancer Prev. 2016;17(7):3483-3487. 27509996

[17] Tobaiqy M, Thomas D, MacLure A, MacLure K. Smokers’ and non-smokers’ attitudes towards smoking cessation in Saudi Arabia: a systematic review. Int J Environ Res Public Health. 2020;17(21):8194. doi:10.3390/ijerph17218194 33171946 PMC7664210

